# Antimicrobial Potential of Bacteriophages JG005 and JG024 Against *Pseudomonas aeruginosa* Isolates from Canine Otitis

**DOI:** 10.3390/vetsci12070646

**Published:** 2025-07-07

**Authors:** Maura R. Lourenço, Eva Cunha, Luís Tavares, Manuela Oliveira

**Affiliations:** 1 CIISA—Centre for Interdisciplinary Research in Animal Health, Faculty of Veterinary Medicine, University of Lisbon, 1300-477 Lisbon, Portugal; maura.h.r.lourenco@gmail.com (M.R.L.); ltavares@fmv.ulisboa.pt (L.T.); moliveira@fmv.ulisboa.pt (M.O.); 2 Associate Laboratory for Animal and Veterinary Sciences (AL4AnimalS), 1300-477 Lisbon, Portugal; 3 cE3c—Centre for Ecology, Evolution and Environmental Changes & CHANGE—Global Chance and Sustainability Institute, Faculty of Sciences, University of Lisbon, 1749-016 Lisbon, Portugal

**Keywords:** antimicrobial resistance, bacteriophage, biofilm, dog, otitis externa, *Pseudomonas aeruginosa*

## Abstract

Canine otitis externa caused by *Pseudomonas aeruginosa* is a relevant disease in veterinary medicine. Given *P. aeruginosa’s* high priority status for the development of new antimicrobials, innovative strategies like bacteriophage therapy are essential. Lytic bacteriophages are viruses with high specificity for their bacterial hosts, making them a promising therapeutic choice in both human and veterinary medicine. This study aimed to evaluate the antimicrobial potential of bacteriophages JG005 and JG024, first characterized in terms of their biofilm-forming ability and antimicrobial susceptibility profile, against *P. aeruginosa* isolates obtained from dogs with otitis externa,. Bacteriophages titer, host range, and activity were assessed against *P. aeruginosa* biofilms via microtiter assays using crystal violet and Alamar Blue. JG024 showed lytic activity against 61.2% (n = 30/49) of the isolates, while JG005 showed lytic activity against 38.8% (n = 19/49) of the isolates. Crystal violet quantification showed that JG005 can promote strong microbial suppression of 60% (n = 6/10) and 50% (n = 5/10) of the isolates at a multiplicity of infection (MOI) of 10 and 100, respectively. JG024 presented strong microbial suppression of 20% (n = 2/10) of the isolates regardless of the MOI level tested. These phages show promising potential as an innovative treatment for canine otitis externa caused by *P. aeruginosa*, but further studies are needed before future clinical use.

## Simple Summary

*Pseudomonas aeruginosa* is a major concern in veterinary medicine due to its antimicrobial resistance and biofilm-forming ability, particularly in canine otitis externa. This study explored the potential of bacteriophages as a therapeutic approach to this disease. Two phages, JG005 and JG024, were tested against *P. aeruginosa* isolates obtained from dogs with otitis externa. The isolates were first characterized in terms of biofilm production and antibiotic resistance profile. Phage activity was then evaluated for biofilm reduction. JG024 was effective against 61.2% of the isolates, while JG005 showed activity against 39%. JG005 also achieved higher biofilm reduction than JG024. These results highlight the potential of bacteriophages as a promising alternative for treating canine ear infections, reinforcing the need for further studies.

## 1. Introduction

*Pseudomonas aeruginosa* is a Gram-negative rod with a worldwide distribution [1]. They are opportunistic pathogens of animals, humans, and plants, being related to several infections, both in human and veterinary medicine [1]. *P. aeruginosa* is particularly difficult to eliminate because it can express multiple virulence factors, including biofilm formation, and frequently presents resistance to various antibiotics. As a result, it is able to cause infections in animals undergoing antibiotic treatment, as well as in immunocompromised hosts [1,2,3,4,5,6]. The pathogen *P. aeruginosa* is described by the World Health Organization as a high priority for research and for the development of strategies aimed at preventing and controlling the spread of antimicrobial resistance [7]. These strategies should consider that the ability of this pathogen to produce biofilms may prevent antibiotics from reaching bacterial cells, and that the biofilm itself may include multidrug-tolerant persister cells that can withstand antibiotic attack, resulting in prolonged and recurrent infections [8,9].

Otitis externa (OE) is an inflammation of the external ear canal [10]. It is a common disease in dogs, with a prevalence ranging from 10% to 20% [11], being classified accordingly to its etiology. Perpetuating and predisposing factors may contribute to ear disease, preventing disease resolution and leading to recurrence if the appropriate treatment is not provided [12]. Environmental species, like *Pseudomonas* spp., and commensal organisms, like staphylococci and *Malassezia* spp., are frequent secondary agents associated with OE [13]. As previously stated, *P. aeruginosa* is able to form biofilms, which may contribute to the chronicity and recurrence of otitis externa [14].

The search for alternative treatment strategies is important as the discovery of new antibiotic classes has slowed down in the last decades. Several non-conventional therapeutics for *P. aeruginosa* infections have shown promising results when applied alone or in combination with conventional therapies, including quorum-sensing systems inhibitors; the application of bacterial lectins; the use of iron chelators, vaccines, and nanoparticles; and bacteriophage therapy [15].

Bacteriophages are obligatory intracellular viruses that infect bacteria [16,17]. Most phages reported in the scientific literature are dsDNA tailed phages of the *Caudovirales* order [18]. Phages are categorized according to their life cycle as lytic or virulent, lysogenic or temperate, or pseudo-lysogenic [19]. Lytic phages are the best choice for therapeutic use because they cause bacterial cell lysis without being integrated into the host DNA, and, therefore, they do not promote resistance transfer [18]. Moreover, bacteriophages are good candidates for antimicrobial therapy as they are active against Gram-positive [20] and Gram-negative bacteria, including multidrug-resistant pathogens [21]; they have high specificity, as they do not affect eukaryotic cells [22], reducing the risk of side effects; and they possess self-reproducing capability if the bacterial host is present [16]. In addition, they have a wide distribution upon systemic administration, a potential anti-inflammatory effect, they are cost effective, and present improved efficacy when compared with antibiotics [16].

Considering the advantages of the use of bacteriophages for the treatment of infections caused by resistant bacteria, this study aimed to evaluate the antimicrobial and antibiofilm potential of the bacteriophages JG005 (DSM 19872) and JG024 (DSM 22045) against established biofilms formed by *P. aeruginosa* isolates obtained from the ear canal of dogs with otitis externa.

## 2. Materials and Methods

### 2.1. Bacterial Strains

A collection of 49 *P. aeruginosa* clinical isolates obtained between 2016 and 2021 from the ear canal of dogs with otitis externa, belonging to the Laboratory of Microbiology and Immunology in the Faculty of Veterinary Medicine at the University of Lisbon, Portugal, was used in this study. The reference strains *P. aeruginosa* ATCC^®^ 27853™, *P. aeruginosa* DSM 19880, *P. aeruginosa* DSM 19882, and *Escherichia coli* ATCC^®^ 25922™ were also tested as controls. All isolates were propagated in brain–heart-infusion agar (VWR, Leuven, Belgium), an enrichment medium, and purity was confirmed through morphological evaluation of the bacterial colonies and microscopic observation after Gram staining. Throughout the study, the isolates were stored at −20 °C in a peptone water solution (VWR, Leuven, Belgium) supplemented with 20% glycerol (VWR, Leuven, Belgium). For the experiments, bacterial cultures were grown in brain–heart-infusion broth (VWR, Leuven, Belgium) or Luria–Bertani broth (LB, VWR, Leuven, Belgium), and incubated for 24 h at 37 °C.

### 2.2. Evaluation of the Biofilm-Forming Ability of the P. aeruginosa Isolates

Isolates’ ability to produce biofilm was evaluated using Congo Red agar, composed of Brain–Heart-Infusion-agar medium (VWR, Leuven, Belgium) supplemented with sucrose (Milipore, Burlington, MA, USA) at 5% and Red Congo dye (Sigma-Aldrich, Saint Louis, MO, USA) at 0.0008% [23,24]. Reference strains *P. aeruginosa* ATCC© 27853™ and *E. coli* ATCC© 25922™ were used as positive and negative controls for biofilm production, respectively. The isolates were inoculated in Congo Red agar and incubated at 37 °C for 72 h, with colony morphology being evaluated after 24, 48, and 72 h of incubation. Isolates that originated as black or darkened colonies with a dry consistency and crystalline appearance were classified as biofilm-producers, while isolates that originated as reddish colonies were classified as non-producers [23,24].

### 2.3. Evaluation of Antimicrobial Susceptibility Profile of the P. aeruginosa Isolates

The isolates’ antimicrobial susceptibility profile was determined using the disk diffusion technique [25] and the guidelines of the Clinical and Laboratory Standards Institute [26]. The antimicrobials evaluated were selected based on their relevance to veterinary medicine, and included: amikacin (30 μg, Fisher Scientific, Waltham, NH, USA), carbenicillin (100 μg, Fisher Scientific, NH, USA), ceftazidime (30 μg, Fisher Scientific, NH, USA), ciprofloxacin (5 μg, Fisher Scientific, NH, USA), enrofloxacin (5 μg, Fisher Scientific, NH, USA), gentamicin (10 μg, Fisher Scientific, NH, USA), marbofloxacin (5 μg, Mast Group, Liverpool, UK), ofloxacin (5 μg, Fisher Scientific, NH, USA), piperacillin (100 μg, Fisher Scientific, NH, USA), streptomycin (10 μg, Fisher Scientific, NH, USA), and tobramycin (10 μg, Fisher Scientific, NH, USA). Moreover, 10% of replicates were performed on independent days.

Results allowed the identification of multidrug-resistant isolates [27] and the determination of the multiple antibiotic resistance (MAR) index [28,29], which allows us to assess the level of resistance of bacterial isolates to several antibiotics. Specifically, a MAR index of 0.20 or higher suggests that the isolates originated from high-risk environments, potentially indicating a greater risk of infection [29]. MAR index value was determined using the following formula:

$$\text{MAR index}=\frac{\mathrm{number of antimicrobials to which isolates were resistant}}{\mathrm{number of antimicrobials tested}}$$

### 2.4. Preparation of a Bacteriophage Stock

Bacteriophages JG005 (DSM 19872) and JG024 (DSM 22045) were acquired from the Leibniz Institute DSMZ (Braunschweig, Germany) as phage suspensions with titers ranging from 1 × 10^8^ to 1 × 10^11^ PFU/mL. These phages were previously characterized as lytic phages. Moreover, they are dsDNA bacteriophages, with an icosahedral head and a contractile tail, genomically classified within the genus *Pbunavirus*, the family Myoviridae, and the class Caudoviricetes. Regarding growth ability, JG024 exhibits a burst size of approximately 180 phage progeny per infected cell [30,31,32,33,34,35].

Bacteriophage propagation was performed using the reference strains *P. aeruginosa* DSM 19880 and DSM 19882 as specific hosts for bacteriophages JG005 and JG024, respectively [36,37,38]. Bacteriophages propagation was performed using two methods: the adsorption method [37] and the spot method [38].

### 2.5. Titration of Bacteriophages

The titer of the bacteriophages suspensions was determined through a double agar overlay plaque assay, following the protocol described by [39], using a saline magnesium buffer composed of 0.1 M sodium chloride (NaCl, Merck, Rahway, NJ, USA 1.06404.1000), 8 mM magnesium sulphate (MgSO4, Labkem, Baldoyle, Ireland, MGSU-07A-500), and 50 mM Tris Hydrochloride (Tris-HCL, Sigma-Aldrich, Saint Louis, MO, USA), adjusted to pH 7.5. Briefly, bacteriophages suspensions were 10-fold diluted in saline magnesium buffer [38]. *P. aeruginosa* DSM 19880 and DSM 19882 were propagated in LB agar (VWR, Leuven, Belgium) for 24 h at 37 °C. After incubation, a bacterial suspension with 0.5 McFarland was prepared in 0.9% saline solution. Afterwards, 100 μL of all phage’s suspensions and 100 μL of the respective bacterial suspension were transferred to 3 mL LB medium supplemented with 0.75% agar, mixed and poured over the surface of an LB agar plate. After solidification of the agar overlayers, plates were incubated for 24 h at 37 °C, after which phage plaques on the agar plates that presented between 30 to 300 phage plaques were counted.

To determine the titer of the phage preparation, the following formula was used [39]:

Number of plaques×10×reciprocal of counted dilution=PFU/mL

The determination of bacteriophage suspensions titers was performed in duplicate.

### 2.6. Bacteriophage Host Range

The two bacteriophages were tested against the 49 *P. aeruginosa* clinical isolates using a modification of the bacteriophage spot-on-lawn test [40]. Isolates were propagated on LB agar medium and incubated for 24 h at 37 °C. After incubation, a bacterial suspension in 0.9% saline solution with 0.5 McFarland was prepared. Then, 200 μL of this suspension was inoculated in 3 mL of LB supplemented with 0.7% agar and poured over LB agar plates. The top agar layer was allowed to solidify, after which 5 μL of each bacteriophage suspension, with approximately 10^8^ PFU/mL, were spotted on the bacterial lawns formed by each isolate. Then, the plates were incubated for 24 h at 37 °C, after which they were observed to detect the presence or absence of bacterial lysis. Specific bacteriophage-susceptible isolates showed clear areas in the areas in which the bacteriophage suspensions were spotted. Results were used to classify the phages host range according to [41], which considers that a broad-spectrum phage is a phage that has a wide host range, associated with a susceptibility range superior to 50%.

### 2.7. Bacteriophage Activity Against Established Biofilms

The activity of the two bacteriophages against established biofilms was determined using a modified protocol based on [42,43,44]. Briefly, the isolates susceptible to the bacteriophages and the reference strains *P. aeruginosa* ATCC^®^ 27853™, *P. aeruginosa* DSM 19880, and *P. aeruginosa* DSM 19882 were propagated in LB agar for 24 h at 37 °C. After incubation, a bacterial suspension with 0.5 McFarland was prepared in 0.9% saline solution. This suspension was diluted in Tryptic Soy Broth (TSB) (VWR, Leuven, Belgium) supplemented with 0.25% glucose until it reached ≈ 1–2 × 10^6^ CFU/mL. Afterwards, 100 μL of each bacterial suspension was added to a 96-well flat-bottomed polystyrene microtiter plate and incubated for 24 h at 37 °C, to allow biofilm formation. After incubation, planktonic bacteria were carefully removed with a sterile pipette and 150 μL of individual bacteriophage suspensions diluted in TSB supplemented with 0.25% glucose was added to the wells using the following multiplicities of infection (MOI): 10 (10^7^ PFU/mL) and 100 (10^8^ PFU/mL). Then, the microplates were incubated for 24 h at 37 °C, after which the presence of biofilm was evaluated using Alamar Blue (AB), according to [43], and 0.25% Hucker crystal violet, according to [45]. The following controls were included in all assays: media alone, media plus Alamar Blue or crystal violet plus bacteriophage suspension, and bacteria suspension plus media plus Alamar Blue or crystal violet.

According to [43], after incubation, 5 μL of AB was added to the wells of the microtiter plate, followed by gentle agitation at 100 rotations per minute and incubation for 1 h at 37 °C. Then, plates were gently shaken again, and absorbance at 570 nm and 600 nm was determined using a FLUOstar OPTIMA microplate reader (BMG LABTECH, Ortenberg, Germany).

The percentage of growth inhibition of the isolates by each bacteriophage was calculated using the following formula [43]:

$$100-\left(\frac{\left({ɛ}_{ox}\right)\lambda_{2}A\lambda_{1}-\left({ɛ}_{ox}\right)\lambda_{1}A\lambda_{2}}{\left({ɛ}_{red}\right)\lambda_{2}A{\prime \lambda }_{1}-\left({ɛ}_{red}\right)\lambda_{1}A\lambda_{2}}\times 100\right)$$

where ${ɛ}_{ox}$ = molar extinction coefficient of Alamar Blue oxidized form (blue), ${ɛ}_{red}$ = molar extinction coefficient of Alamar Blue reduced form (pink), A = absorbance of test wells, A’ = absorbance of positive control well, $\lambda_{1}$ = absorbance at 570 nm, and $\lambda_{2}$ = absorbance at 600 nm.

According to [45], after incubation, the content of all wells of the microplate was carefully aspirated to eliminate planktonic forms, and the wells were washed three times at room temperature with phosphate-buffered saline at pH 7.0. Then, the microtiter plate was incubated in an inverted position for 1 h at 60 °C, allowing the fixation of adherent cells. Afterwards, 150 μL of 0.25% crystal violet was added to the wells, followed by incubation at room temperature for 5 min. The stain excess was aspirated, and the microtiter plate rinsed with water. After air-drying at room temperature, 150 μL of 95% ethanol was added to each well of the microtiter plates. Plates were then covered with a lid and incubated at room temperature for 30 min. After incubation, the optical density (OD) of the wells was evaluated at 600 nm (OD _600_) by horizontal bidirectional reading using a microplate reader. The percentage of growth inhibition of the isolates by each bacteriophage was calculated using the following formula [46]:

$$100-(\frac{{OD}_{600}Phage\;treated\;wells}{{OD}_{600}Controls\;without\;phage\;treatment}\times 100)$$

Three replicas were performed for each biofilm susceptibility experiment, in three independent assays.

Results allowed us to classify the level of antimicrobial suppression of each phage, according to [44], which states that a strong antimicrobial suppression can be defined as a reduction in Alamar Blue or crystal violet equal or greater than 50% in comparison with the positive control.

### 2.8. Statistical Analysis

Statistical analysis was performed using Microsoft Excel (Microsoft Corporation, Redmond, WA, USA). Quantitative variables were presented as mean values ± standard deviation, along with the minimum and maximum values. The MAR index was calculated, as previously described in the Section 2.3. The evaluation of the isolates’ biofilm-forming ability, determined using either Alamar Blue or crystal violet, was conducted following the formulas outlined in Section 2.7. Biofilm reduction was determined by comparison with the positive control (wells containing biofilm not subjected to any treatment). The Shapiro–Wilk test was performed to assess the normality of data distribution. Subsequently, the Wilcoxon Rank Sum test was applied to compare the percentage of reduction in the absorbance/optical density values obtained in the crystal violet and Alamar Blue assays. A *p* value < 0.05 was considered as statistically significant.

## 3. Results

### 3.1. Evaluation of the Biofilm-Forming Capacity of the Isolates Under Study

The evaluation of the isolates’ biofilm-forming ability in Congo Red agar revealed that 38.8% (n = 19/49) of the isolates were biofilm-producers. From these, 10 isolates were able to produce biofilm after 24 h, 7 isolates after 48 h, and the remaining 2 only demonstrated this ability after 72 h of incubation.

### 3.2. Evaluation of the Isolates’ Antimicrobial Susceptibility Profile

**Figure 1:**
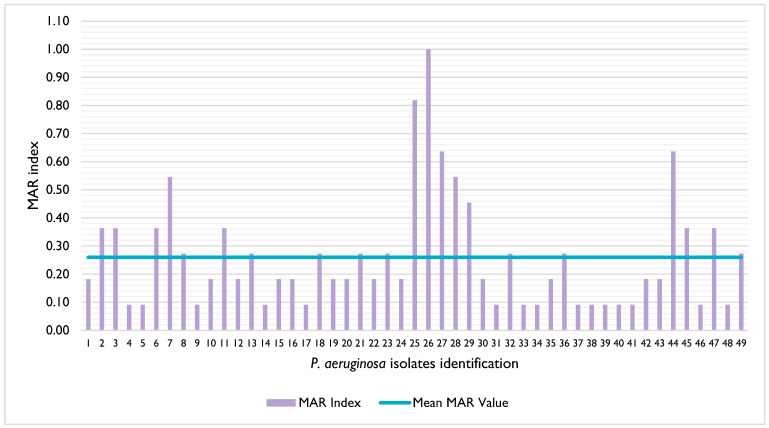
Distribution of the multiple antibiotic resistance (MAR) index of the *P. aeruginosa* isolates under study. The mean MAR value is represented by the blue line.

The isolates’ resistance rates ranged from 0% (ciprofloxacin) to 44.9% (streptomycin) (Table 1). Moreover, 4.1% (n = 2/49) of the isolates were susceptible to all antibiotics tested, 2.0% (n = 1/49) were non-susceptible to all antibiotics tested, and 51.0% (n = 25/49) of the isolates were classified as multidrug resistant, according to [27] (Supplementary File S1). The MAR index of the isolates ranged between 0.09 and 1, with a mean value of 0.26 (Figure 1); in addition, 42.9% (n = 21/49) of the isolates presented a MAR index superior to 0.2 [28].

### 3.3. Titration of Bacteriophages

The adsorption method [37] allowed us to obtain bacteriophage titers ranging from 10^8^ to 10^10^ PFU/mL for bacteriophage JG005 and from 10^9^ to 10^11^ PFU/mL for bacteriophage JG024, while with the spot-on method [38], the titers ranged from 10^7^ to 10^10^ PFU/mL for bacteriophage JG005 and from 10^6^ to 10^8^ PFU/mL for bacteriophage JG024.

### 3.4. Bacteriophage Host Range

Regarding the bacteriophage host range, it was possible to observe that 38.8% (n = 19/49) of the isolates were susceptible to bacteriophage JG005, while 61.2% (n = 30/49) were susceptible to bacteriophage JG024; therefore, bacteriophage JG005 has a narrower host range than JG024.

Regarding biofilm-positive isolates, 21.1% (n = 4/19) of these isolates were susceptible to bacteriophage JG005 and 21.1% (n = 4/19) were susceptible to bacteriophage JG024, with 21.1% (n = 4/19) of the biofilm producer isolates being susceptible to both bacteriophages. Moreover, 36.8% (n = 7/19) of these isolates were not susceptible to either of the two bacteriophages tested.

### 3.5. Bacteriophage Activity Against Established Biofilms

**Figure 2:**
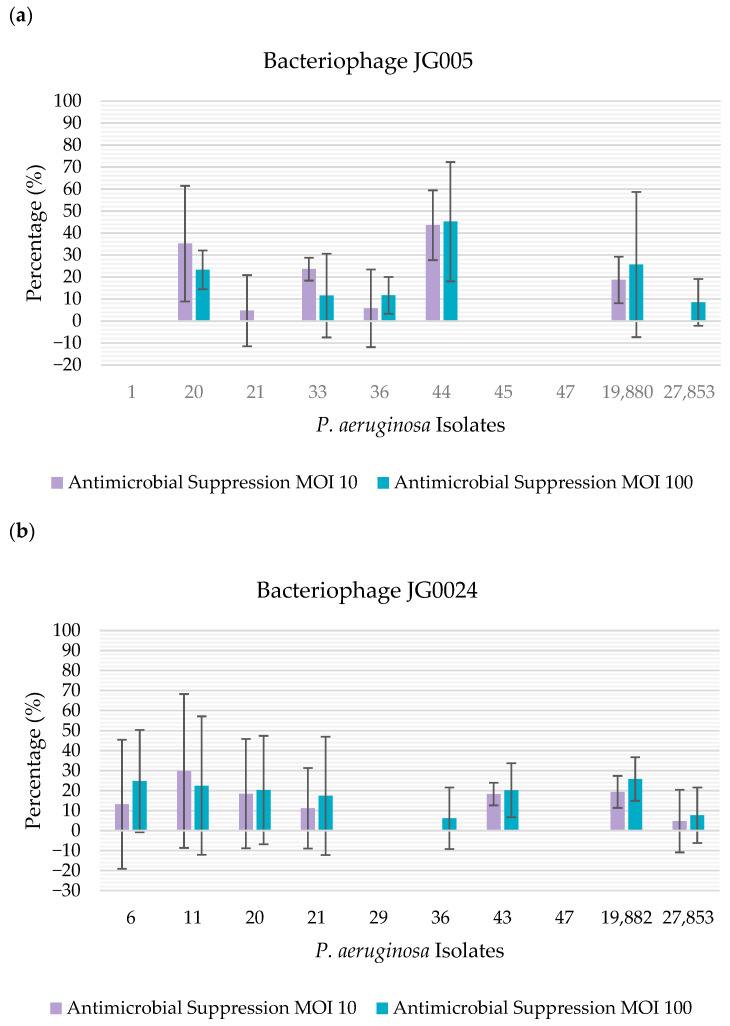
Percentage of antimicrobial suppression by the bacteriophages, determined using Alamar Blue: (**a**) percentage of antimicrobial suppression by bacteriophage JG005. The percentage of antimicrobial suppression is represented on the y-axis. The isolates tested are represented on the x-axis; (**b**) percentage of antimicrobial suppression by bacteriophage JG024. The percentage of antimicrobial suppression is represented on the y-axis. The isolates tested are represented on the x-axis.

The bacteriophage activity against established biofilms was evaluated using a total of 12 biofilm-positive isolates that were susceptible to at least one of the two phages, allowing us to use 10 isolates to test each bacteriophage. When using Alamar Blue as a method of quantification of biofilm suppression, it was possible to observe that bacteriophage JG005 showed antibiofilm activity against 50% (n = 5/10) of the isolates at both MOI levels tested; however, when evaluating each MOI individually, the phage was able to suppress 60% (n = 6/10) of the isolates at MOI 10 and 60% of the isolates at MOI 100. On the other hand, bacteriophage JG024 showed activity towards 70% (n = 7/10) of the isolates at MOI 10, and against 80% (n = 8/10) of the isolates at MOI 100 (Figure 2).

**Figure 3:**
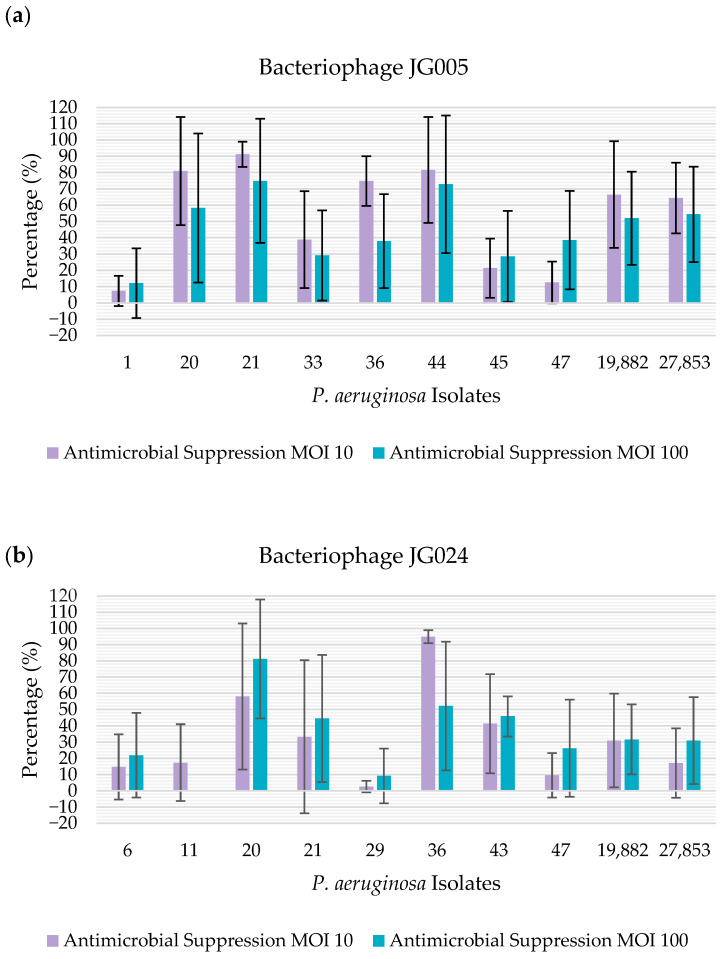
Percentage of antimicrobial suppression of the isolates by the bacteriophages, determined using crystal violet: (**a**) percentage of antimicrobial suppression of the isolates by bacteriophage JG005. The percentage of antimicrobial suppression is represented on the y-axis. The isolates tested are represented on the x-axis; (**b**) percentage of antimicrobial suppression of the presented isolates by bacteriophage JG024. The percentage of antimicrobial suppression is represented on the y-axis. The isolates tested are represented on the x-axis.

On the other hand, when using crystal violet as a method of quantification of biofilm suppression, bacteriophage JG005 showed antibiofilm activity towards 100% (n = 10/10) of the isolates at both the MOI levels tested. Regarding bacteriophage JG024, this phage showed antibiofilm activity towards 90% (n = 9/10) of the isolates at both the MOI levels tested; more specifically, the phage was able to suppress 100% (n = 10/10) of the isolates at MOI 10 and 90% (n = 9/10) of the isolates at MOI 100 (Figure 3).

In this study, using Alamar Blue as a method of quantification of biofilm suppression and considering the mean value of absorbance for each strain evaluated, it was not possible to detect a strong antimicrobial suppression, regardless of the bacteriophage and MOI used.

Using crystal violet, it was possible to observe that bacteriophage JG005 presented a strong antimicrobial action regarding 60% (n = 6/10) of the isolates at MOI 10, and towards 50% (n = 5/10) of the isolates at MOI 100. For bacteriophage JG024, a strong antimicrobial suppression was obtained regarding 20% (n = 2/10) of the isolates, regardless of the MOI.

The ability of the two methods to quantify biofilm suppression by the two phages was compared through statistical analysis. As the data did not follow a normal distribution, the Wilcoxon Rank Sum test was used to compare the percentage of reduction in the optical density and absorbance values obtained in the crystal violet and Alamar Blue assays, respectively. For bacteriophage JG024, no statistically significant difference (*p* value < 0.05) was observed between the two methods used for quantifying antimicrobial suppression in *P. aeruginosa* biofilms, regardless of the MOI applied. In contrast, when comparing the overall results for bacteriophage JG005, a statistically significant difference was detected between the two methods. When analyzing the results for each MOI separately, a statistically significant difference was found at MOI 100, with higher suppression being observed when using the crystal violet method, whereas no significant difference was observed at MOI 10.

## 4. Discussion

### 4.1. Evaluation of the Biofilm-Forming Capacity of the Isolates Under Study

Biofilm production promotes the enclosure of bacterial communities in a self-producing matrix, mainly constituted of exopolysaccharides [24,47]. In the case of otitis, biofilms impair the cleaning of the ear canal and the penetration of antimicrobial compounds [47]. The evaluation of the biofilm-forming ability of the isolates under study was performed using Congo Red agar as this method has the advantages of being rapid, sensible, and presenting good reproducibility [23,24,48]. Moreover, *P. aeruginosa* can produce an exopolysaccharide matrix whenever environmental conditions are suitable for bacterial colonization, unlike some bacterial species that need specific pH or nutritional conditions to form biofilms, as observed for some strains of *E. coli* that need casamino acid supplementation to grow [49,50]. In this study, 38.8% of the isolates presented the ability to produce biofilm. This is in agreement with the results from a study by [51], in which 41.8% of the isolates were biofilm producers. The high percentage of biofilm-producing strains obtained shows that their detection is especially relevant for assessing the virulence potential of the pathogens responsible for otitis and for establishing adequate therapeutic protocols [48].

### 4.2. Evaluation of Antimicrobial Susceptibility Profile of the Isolates Under Study

The isolates under study were characterized in terms of their susceptibility against a panel of eleven antimicrobials. The highest resistance rate observed was towards streptomycin, which is in accordance with the study by [52], followed by carbenicillin, enrofloxacin, and marbofloxacin. Comparable results were described in other reports [53,54,55,56,57].

From the antibiotics tested, gentamicin and marbofloxacin are the only aminoglycoside and fluoroquinolone, respectively, available in Portugal for topical use in canine otitis externa [58]. The detection of isolates resistant to these antibiotics raises concerns about their careful and rational use in veterinary medicine, pointing to the relevance of carrying out antibiotic susceptibility tests prior to the establishment of treatment protocols for canine otitis externa.

Regarding multidrug resistance, 51.0% of the isolates were classified as multidrug-resistant, a higher percentage than the one described by [57] (40%), [51] (28.4%), and [52] (2.8%), but much lower than the one reported by [59] and [60] (92%). Otitis externa in dogs is considered to be a source of infection to humans through direct or indirect contact [61]. Given the close interactions between owners and their dogs [60], the high percentage of multidrug-resistant *P. aeruginosa* isolates obtained may represent a zoonotic issue due to the potential spread of multidrug-resistant strains [60,62]. Therefore, antimicrobial susceptibility testing of *P. aeruginosa* associated with otitis externa is an important step in the selection of appropriate therapy [63,64,65]. This is especially relevant considering that almost half of the isolates presented a MAR index superior to 0.2, which may indicate a higher risk of infection [28]. Similar results were observed by other researchers [51].

### 4.3. Bacteriophage Host Range

Bacteriophage therapy focuses on the potential use of phages to treat bacterial infections, and is receiving new attention due to the rise in infections caused by multidrug-resistant bacteria [66].

To the author’s best knowledge, this is the first study in which the host range of bacteriophages JG024 and JG005 was determined using *P. aeruginosa* isolates obtained from dogs with otitis externa. A study by [31], based on 19 *P. aeruginosa* isolates from patients with cystic fibrosis, demonstrated that bacteriophage JG024 was able to infect 84% of all the clinical isolates tested, promoting clear lysis in 68% of the isolates. The authors also tested the ability of this bacteriophage to infect 100 *P. aeruginosa* environmental strains, and found that it was able to promote clear lysis in 45% of the strains [31]. Also, to the author’s best knowledge, there are no studies available on the evaluation of the host range of the bacteriophage JG005 concerning *P. aeruginosa* clinical isolates of animal origin. Other researchers have evaluated the host range of distinct *Myoviridae* phages against *P. aeruginosa* isolates from distinct origins and described host range values varying from 30 to 52.6% [32,67,68,69,70].

Additionally, a study by [71] reported that bacteriophage JG005 lysed 70% of the environmental strains tested, while bacteriophage JG024 lysed 49%; moreover, approximately 55% of the strains were lysed by both bacteriophages.

The determination of a phage’s host range is a critical step for using a specific bacteriophage for therapy [72]. Results from this study show that phage JG024 can be classified as a broad-spectrum phage, having lytic ability against 61.2% of the isolates, while phage JG005 presented a narrower host range, being only effective against 38.8% of the isolates. Phages with a wider host range are better suited for therapy [72] as they have a higher potential of infecting several emerging pathogenic strains, whereas lytic phages with a narrow host range may require the use of phage cocktails, making the phage propagation, storage, and clinical development prohibitively expensive [73]. We acknowledge that a limited host range is a significant limitation for the therapeutic application of bacteriophages. As demonstrated in our study, phage JG005 exhibited a relatively narrow host range compared to JG024, which may restrict its clinical application. This limitation supports the need of developing phage cocktails or alternative strategies to ensure effective targeting of diverse bacterial strains, highlighting a key challenge in advancing phage therapy.

### 4.4. Bacteriophage Activity Against Established Biofilms

Considering the potential association between biofilm-formation and bacterial resistance, it is of major importance to assess bacteriophage activity against established biofilms. Biofilm formation can be evaluated using chemical techniques based on dyes or fluorochromes that can bind to or adsorb to several biofilm components [74]. In this study, two different dyes were used to evaluate the effect of phages on *P. aeruginosa* biofilms, namely Alamar Blue and crystal violet.

The crystal violet staining method for biofilm quantification using microtiter plates continues to be one of the most often used methods [75]. This stain binds to the bacterial cellular components [76], especially in the initial stages of biofilm formation, enabling the optical visualization of biofilm thickness and the measurement of total biofilm biomass. However, since the extracellular DNA, polysaccharides, and protein matrix are also stained by crystal violet, results may not be directly correlated with the quantity of living bacteria in the biofilm [76]. On the other hand, Alamar Blue is a redox indicator that can be used to quantify biofilms [77] and assess bacterial viability [78]. The chemical reduction in Alamar Blue occurs as a result of the metabolic activity of bacteria, promoting fluorescence and color changes, with the extent of the color change depending on the cells’ viability level [43].

To the author’s best knowledge, this is the first report on the in vitro evaluation of the antimicrobial efficacy of bacteriophages JG024 and JG005 against *P. aeruginosa* biofilms formed by clinical isolates of animal origin using both crystal violet and Alamar Blue. Overall, no statistically significant differences were observed between the two methods used to evaluate biofilm suppression. Differences were detected only for bacteriophage JG005 at a MOI of 100, with the crystal violet method allowing us to detect a higher suppression. Other researchers have used crystal violet to evaluate the antimicrobial effect of distinct phages against *P. aeruginosa* biofilm cells and observed a biomass reduction ranging from 44 to 90% [79,80,81]. On the other hand, using Alamar Blue, [80] observed a higher suppression (79%) of the metabolic activity of biofilm-based cells after 8 h of exposure to phages, followed by a significant recovery of the metabolic activity after 24 h of treatment [80].

In the future, studies aiming to increase the phages’ host range should be performed [82] to increase the efficacy of bacterial suppression [83], and to decrease the development of phage resistance [84]. Bacteriophages have the advantage of penetrating the inner layers of the biofilms. Moreover, they can dissolve the biofilm matrix by producing matrix-degrading enzymes, such as polysaccharide depolymerases, or by inducing the bacterial hosts to secrete enzymes that also degrade the extracellular polymeric substances (EPS), thus impairing biofilm formation and promoting its breakdown [85]. Additionally, bacteriophages are capable of infecting persister cells and eliminating them upon reactivation. They can also produce enzymes that inhibit biofilm formation [85]. In addition, phages can also destroy biofilms indirectly by killing bacteria before attaching, in their planktonic form, or after surface colonization [86].

The use of bacteriophages for the treatment of chronic *P. aeruginosa* otitis externa in dogs has already been reported in three studies. In fact, [87] described for the first time the use of phage therapy to treat a *P. aeruginosa* infection in a dog, namely a 5-year-old Saint Bernard with chronic otitis externa, after the failure of treatment with repeated courses of topical and systemic antimicrobials. They reported an improvement of the ear condition and a complete recovery 9 months after bacteriophage application with no adverse reactions [87]. Also, [88] performed a clinical trial using a cocktail of six bacteriophages administered to ten dogs with *P. aeruginosa* otitis externa. The authors observed that, 48 h after cocktail application, there was a 30% mean reduction in the otitis clinical score, a 67% reduction in the bacterial count, and an increase in the bacteriophage count [88]. Additionally, in 2025, [89] tested a phage cocktail in five dogs, all with chronic otitis externa, demonstrating a significant reduction in bacterial counts and notable clinical improvements [89]. Curiously, in 2020, a study conducted in the United Kingdom evaluated the veterinarians’ and pet owner’s acceptance of the use of phage therapy in companion animals. After a brief explanation of what phages are and how phage therapy works, a total of 75% of the participants positively supported the use of phage therapy for the treatment of infections in companion animals. Even though veterinarians were more familiar with the concept, pet owners stated that they would trust the veterinarians’ advice, reinforcing the potential and viability of phage therapy in veterinary medicine [90].

Phages represent a promising alternative therapy and should be considered in research and development projects aimed at discovering new antimicrobial compounds. Studies on bacteriophage activity, particularly against biofilm-associated cells using clinical isolates, are crucial as they provide valuable insights into the current clinical landscape of these isolates, especially when correlated with their resistance profiles. Our study demonstrated that phages JG024 and JG005 are capable of infecting the *P. aeruginosa* isolates associated with canine otitis, including multidrug-resistant strains, in both planktonic and biofilm forms. A total of 49 canine *P. aeruginosa* isolates were analyzed in this study, representing a collection obtained during 5 years, but a larger bacterial collection should be explored in future studies. On the other hand, the fact that this study was conducted exclusively in vitro is also a limitation. Therefore, future studies should evaluate the in vivo efficacy of these phages. It is also important to refer to the fact that the emergence of phage-resistant bacterial pathogens is a key challenge for phage therapy. Notably, resistance to one phage does not necessarily indicate resistance to others, and in some cases it may impose fitness costs on bacteria in the absence of phages [91,92]. To mitigate resistance risks and enhance therapeutic potential, future phage interventions may involve the use of phage cocktails targeting multiple bacterial receptors, leading to the clinical application of bacteriophages in veterinary settings. Other limitations of phage therapy include challenges in regulating phage products and treatments, the potential for host immune responses triggered by the phages, and concerns regarding the route of administration. In the context of otitis treatment, the possibility of topical phage administration reduces the risk of adverse effects, with previous studies showing positive results [87,88]. Nowadays, the first-line treatment for otitis externa includes the topical administration of antibiotics, often combined with anti-inflammatory compounds. Phages have the potential to be successfully utilized in veterinary medicine, likely in combination with other therapies, including anti-inflammatory agents, thereby maximizing treatment effectiveness [89].

## 5. Conclusions

In 2024, the microorganism *P. aeruginosa* was classified by the World Health Organization as one of the high priorities for research and for the development of new antibiotics [7]. It is necessary to find new approaches to treat bacterial pathogens, with phage therapy being a promising complement to antimicrobial therapy [16,93].

The proximity between companion animals and humans makes them a potential focus for the cross-transmission of zoonotic bacteria, including MDR strains [94].

The present study evaluated the inhibitory potential of two phages, JG005 (DSM 19872) and JG024 (DSM 22045), against *P. aeruginosa* isolates obtained from dogs with otitis externa.

Bacteriophage JG024 eliminated 61.2% of the isolates tested, and can be considered a broad-spectrum bacteriophage, in contrast to bacteriophage JG005, which was able to infect only 38.8% of the isolates tested. The activity of the bacteriophages against established biofilms formed by the *P. aeruginosa* canine isolates was evaluated using two different staining techniques. When Alamar Blue was used to assess biofilm suppression, it was not possible to detect a strong antimicrobial suppression, regardless of the bacteriophage and MOI used. On the other hand, when crystal violet was used, it was possible to detect a strong antimicrobial suppression promoted by both bacteriophages and MOIs.

In conclusion, these phages may be considered as an innovative alternative with great potential for the treatment of canine otitis externa caused by *P. aeruginosa*, suggesting the need to continue research aiming at the future use of bacteriophages in clinical settings.

## Figures and Tables

**Table 1:** Antimicrobial susceptibility of the *P. aeruginosa* isolates tested.

Antimicrobial Classes/Antibiotic	Disk Content (µg)	*P. aeruginosa* (n = 49)		
		S (%)	I (%)	R (%)
Aminoglycosides				
Amikacin	30	89.8	4.1	6.1
Gentamicin	10	85.7	2.0	12.2
Streptomycin	10	28.6	26.5	44.9
Tobramycin	10	89.8	4.1	6.1
Penicillins				
Carbenicillin	100	40.8	16.3	42.9
Piperacillin	100	87.8	0.0	12.2
Cephalosporins				
Ceftazidime	30	77.6	10.2	12.2
Fluoroquinolones				
Ciprofloxacin	5	93.9	6.1	0.0
Enrofloxacin	5	18.4	57.1	24.5
Marbofloxacin	5	55.1	22.4	22.4
Ofloxacin	5	87.8	4.1	8.2

Legend: S—susceptible; I—intermediate; and R—resistant.

## Data Availability

The data presented in this study are within this paper and its Supplementary Materials.

## Supplementary Materials

The following supporting information can be downloaded at: https://www.mdpi.com/article/10.3390/vetsci12070646/s1, Supplementary File S1: Characterization of the isolates regarding their antibiotic resistance profile. S: susceptible; I, in bold: intermediate; and R, in bold: resistant.

## Abbreviations

The following abbreviations are used in this manuscript:

LB	Luria–Bertani
MAR	Multiple Antibiotic Resistance
MOI	Multiplicity of Infection
OE	Otitis externa
TSB	Tryptic Soy Broth
